# Theory versus Data: How to Calculate R_0_?

**DOI:** 10.1371/journal.pone.0000282

**Published:** 2007-03-14

**Authors:** Romulus Breban, Raffaele Vardavas, Sally Blower

**Affiliations:** The Semel Institute of Neuroscience and Human Behavior, David Geffen School of Medicine at University of California Los Angeles, Los Angeles, California, United States of America; University of Liverpool, United Kingdom

## Abstract

To predict the potential severity of outbreaks of infectious diseases such as SARS, HIV, TB and smallpox, a summary parameter, the basic reproduction number R_0_, is generally calculated from a population-level model. R_0_ specifies the average number of secondary infections caused by one infected individual during his/her entire infectious period at the start of an outbreak. R_0_ is used to assess the severity of the outbreak, as well as the strength of the medical and/or behavioral interventions necessary for control. Conventionally, it is assumed that if R_0_>1 the outbreak generates an epidemic, and if R_0_<1 the outbreak becomes extinct. Here, we use computational and analytical methods to calculate the average number of secondary infections and to show that it does not necessarily represent an epidemic threshold parameter (as it has been generally assumed). Previously we have constructed a new type of individual-level model (ILM) and linked it with a population-level model. Our ILM generates the same temporal incidence and prevalence patterns as the population-level model; we use our ILM to directly calculate the average number of secondary infections (i.e., R_0_). Surprisingly, we find that this value of R_0_ calculated from the ILM is very different from the epidemic threshold calculated from the population-level model. This occurs because many different individual-level processes can generate the same incidence and prevalence patterns. We show that obtaining R_0_ from empirical contact tracing data collected by epidemiologists and using this R_0_ as a threshold parameter for a population-level model could produce extremely misleading estimates of the infectiousness of the pathogen, the severity of an outbreak, and the strength of the medical and/or behavioral interventions necessary for control.

## Introduction

In Epidemiology, it is essential to quantify the severity of actual (or potential) outbreaks of infectious diseases such as SARS [1], [2], HIV [3], TB [4], and smallpox [5]. The standard procedure is to calculate a parameter called *the basic reproduction number* (R_0_) that characterizes the potential of an outbreak to cause an epidemic. R_0_ has been extensively used to assess transmissibility of pathogens, severity of outbreaks, and epidemiological control [1]–[6]. The established definition of R_0_, as phrased by Anderson and May [6], is “*the average number of secondary infections produced when one infected individual is introduced into a host population where everyone is susceptible*”. They have stated that “*If R_0_ is greater than one then the outbreak will lead to an epidemic, and if R_0_ is less than one then the outbreak will become extinct*” [6]; thus they have assumed that R_0_ is a threshold parameter that establishes whether an outbreak yields an epidemic or not. Here we establish that the average number of secondary infections (i.e., R_0_) is not always an epidemic threshold parameter.

Epidemiologists calculate R_0_ using individual-level contact tracing data obtained at the onset of the epidemic. Once an individual is diagnosed, his/her contacts are traced and tested. R_0_ is then computed by averaging over the number of secondary cases of many diagnosed individuals. This approach is based upon the definition of R_0_, but it does not ensure that the calculated R_0_ is also an epidemic threshold parameter.

Another approach (which is more commonly used) is to obtain R_0_ from population-level data, namely cumulative incidence data. Making certain individual-level modeling assumptions (e.g., the mass-action principle of infectious spread, time independent infection rates, etc.), theorists construct models (typically) based on Ordinary Differential Equations (ODEs) which describe the dynamics of the expected population size in different disease stages without tracking individuals. It is very important to note that the individual-level modeling assumptions cannot be verified using population-level data (i.e., they remain hypothetical). ODE models are formulated in terms of disease transmissibility and progression rates at the population level. These parameters are obtained by fitting the model to population-level data; their relation to the individual-level processes may be quite complex and is generally unknown. Bifurcation analysis of the ODE model yields a threshold parameter [7] that signals the epidemic as indicated by Anderson and May [6] and is formulated in terms of the population-level parameters. This threshold parameter is not usually checked against the value of R_0_ that has been calculated from contact tracing data.

The individual-level and the population-level approaches may produce very different numbers as the first calculates the value of R_0_, whilst the second calculates the value of a threshold parameter. The question of whether the R_0_ obtained by calculating the average number of secondary infections matches the threshold parameter obtained from fitting the epidemiological model to population-level data has been previously studied [8], [9]. In these two papers, the authors show that R_0_ values obtained from different individual-level models (ILMs) do not necessarily agree with those obtained from mean-field ODE models. However, in order to make this point, the modelers consider that the individual-level transmission dynamics occurs on a *social contact network* with a structure that is different from the all-to-all network assumed by ODE models. An infected individual can only infect his/her neighbors in the network which represent a small fraction of the total population. Thus, the R_0_ mismatch can be attributed to the model mismatch. In contrast, in our ILMs, we preserve the assumption that the contact network is all-to-all. However, our research focuses on the *transmission network*. This network is embedded in the social contact network and forms in time during disease spreading by tracking who infected whom. We analyze two distinct ways in which the transmission network can be realized and directly compute R_0_. We thus discuss two distinct ILMs whose prevalence and incidence can be described by an ODE model with an established threshold parameter. We calculate their R_0_ values through the definition and then compare these values with the epidemic threshold parameter. Our results address the question of whether or not an R_0_ (i.e., an average number of secondary infections) can be assigned to an ODE model (which only provides a population-level description of disease propagation) without having any knowledge of the underlying disease transmission network.

## Methods

**Figure 1:**
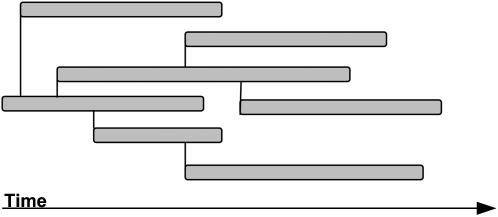
Schematics of a branching process. Two generations of infections are shown. Every horizontal bar segment represents the time interval that a specified individual remains infectious; these time intervals follow a negative exponential distribution with average *μ^−1^*. The time intervals between infections for any given individual follow a negative exponential distribution with an average of *β^−1^*.

A simple ODE model is the Susceptible-Infected (*SI*) model given by *dS/dt = π-βIS/(S+I)* and *dI/dt =  βIS/(S+I)-μI*, where *β* and *μ* are the inflow and, respectively, the outflow of infectious individuals per infectious capita. We apply this model at disease invasion when virtually everyone is susceptible (i.e., *S/(S+I)* is approximately 1) and obtain *dI/dt = βI-μI*. The threshold parameter for the reduced model is *β/μ*; if *β/μ*>1 an outbreak develops into an epidemic, if *β/μ*<1 an outbreak goes extinct. It is important to note that *β* and *μ* are obtained from fitting the model to population-level data, with no clear association to the causal individual-level processes. An individual-level model that is compatible to these dynamics is a branching process; see Fig. 1 and Mathematical Details S1. In this context, *β* is interpreted as the infection rate of an individual and *μ* is the recovery rate of an individual. In this branching process, an individual is expected to infect a number of *β/μ* secondary cases which represents the R_0_ of this ILM. In this case, the average number of secondary infections R_0_ = *β/μ* is also a threshold parameter of the population-level dynamics.

**Figure 2:**
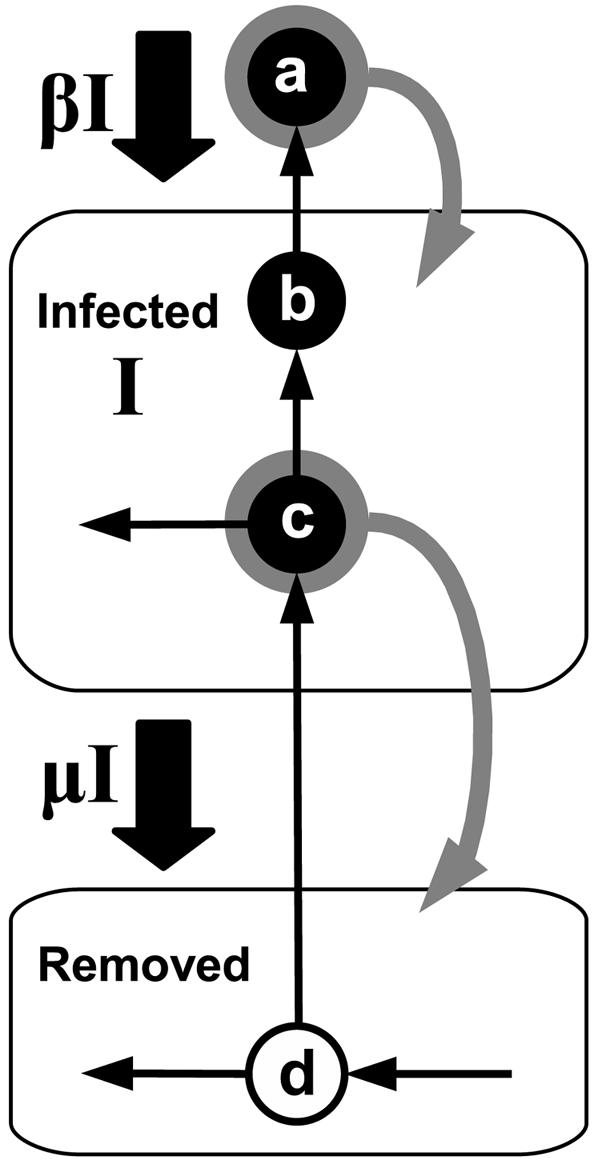
Schematics of our individual-level model (ILM). The model tracks individuals through growing a transmission network by using infection and removal rules [10]. Individuals are represented as the nodes of the network; two individuals a and b are connected by a directed link from b to a if b has infected a. In the Figure, a is a newly infected individual added to the growing transmission network. As an example of an infection rule, a node b is uniformly randomly chosen to be the infectious individual who has infected a. If a removal occurs, an individual c is randomly chosen from the infected group, and is removed. Under the assumption that the number of the secondary infections caused by c is a proxy for the progression of the disease, we choose that the probability that c is removed to be proportional to the number of secondary infections caused by c plus one; i.e., to be proportional to the total number of connections of c. The node c remains connected to the network, but can not cause any new infections; i.e., c becomes the same as node d who previously infected c. The rates of infections and removals per infectious capita are *β* and *μ*, respectively. The branching process presented in Fig. 1 yields the same expected incidence as our ILM which is given by the *SI* ODE model.

**Figure 3:**
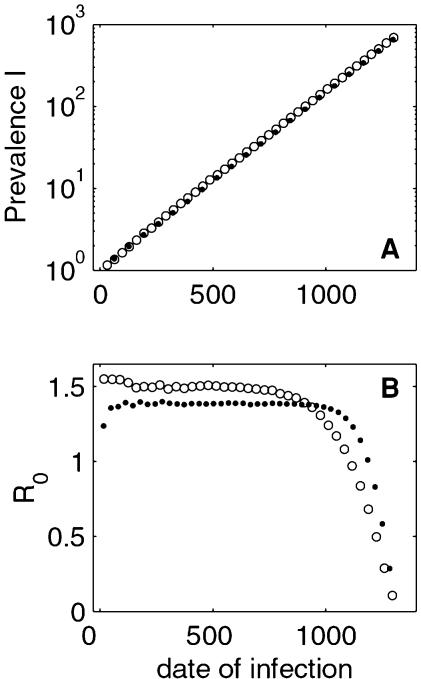
Comparison plots between our example ILM and branching process. A The average prevalence versus time. The open circles represent results for the branching process while the dots represent results for our ILM. We used *β*  = 0.015, *μ*  = 0.01, and we averaged over 1,000 stochastic realizations. On a logarithmic scale, the results fit very well with a straight line, with slope *(β−μ)* and intercept 0, that corresponds to the ODE solution *I(t) = I(*0*)exp[(β−μ)t]*, where *I(*0*) = *1. B The average number of secondary infections stratified versus the date of infection. The open circles represent results for the branching process while the dots represent results for our ILM. We used *β*  = 0.015, *μ*  = 0.01, and we averaged over 15,000 stochastic realizations. The R_0_ of the branching process is 1.5, while the R_0_ of our ILM is approximately 1.4.

However, the branching process is not the only possible ILM that is compatible with the ODE model. Recently, we have shown that other plausible ILMs can be constructed [10] that yield the same ODE dynamics as the *SI* model at disease invasion. We have constructed a new class of ILMs [10]–[12]; see Fig. 2 and Mathematical Details S1. Since, our example ILM generates the same prevalence and incidence as the *SI* ODE model (Fig. 3A) then it would be expected, on the basis of conventional wisdom, to generate the same R_0_. Starting from one infected individual, our simulations integrated the ILM and kept track of the number of secondary infections caused by each individual in the infectious and in the recovered pools. The dynamics were integrated to a certain final time and the collected data were stratified over the date of infection. R_0_ was calculated using the average number of secondary cases generated by infectious individuals, according to the standard definition of Anderson and May [6]. This procedure ensures that each individual included in the calculation of R_0_ is no longer infectious and that there is no right censoring (See Mathematical Details S1). More importantly, it emulates the process of obtaining an R_0_ value by real-world contact tracing data.

## Results

The results (black dots) of the simulation are presented in Fig. 3. For comparison with these results, we present the results (open circles) of a similar simulation for the branching process. The prevalence results for the branching process and the ILM agree very well; see Fig. 3A. For the branching process, R_0_ yields the expected value that agrees with the threshold parameter of the *SI* ODE model; see Fig. 3B. Surprisingly, the graph of R_0_ versus the date of infection plateaus at a lower value than that for the branching model. It is thus evident, as supported by our numerics, that two individual-level models having exactly the same expectations of the corresponding population-level variables (i.e., incidence and prevalence) may yield different R_0_ values (as given by the definition). In the case of our second ILM (see Fig. 2), R_0_ is not the threshold parameter of the *SI* ODE model.

## Discussion

Our results have significant consequences for understanding the concept of R_0_. We explicitly show that certain population-level dynamics, theoretically specified by an ODE model, can be the result of many distinct ILMs. We further demonstrate that the R_0_ obtained from the ILM, by applying the definition of Anderson and May [6], may be different from the epidemic threshold parameter provided by the ODE model. Therefore, population-level predictions based upon an ODE model that use the R_0_ value found by contact tracing as a threshold parameter may be inaccurate.

Our novel results have significant implications for understanding the dynamics of outbreaks of infectious diseases, particularly for the biological understanding of the transmission dynamics of the pathogen, estimating the severity of outbreaks, making health policy decisions, and designing epidemic control strategies. We have shown that the value of R_0_ may not be an accurate measure of the severity of an outbreak since R_0_ may fail to represent an epidemic threshold parameter. Thus, measuring R_0_ through contact tracing (as generally occurs during an outbreak investigation), may not help in predicting the severity of the outbreak and may not be a useful measure for determining the strength of the necessary control interventions. Only an epidemic threshold parameter can be used to design control strategies. This parameter can be obtained through fitting an ODE model to population-level data as mentioned above and will signal epidemic growth whether or not it is equal to the average number of secondary infections. However, obtaining an R_0_ value via contact tracing can be very useful in conjunction with population-level epidemic data to understand the possible transmission mechanisms of the epidemic at the individual level. We thus suggest that the role of R_0_ should be more carefully considered, and that a reevaluation of the role of R_0_ may lead to the development of more effective control strategies.

## Supporting Information

Mathematical Details S1 Here we give more details and references about the individual-level models presented in the main text. We also briefly discuss how the concept of right censoring manifests in our simulations.
(0.05 MB PDF)
