# Supplementary material for: Theory versus Data: How to Calculate R0?
Source: PLoS One. 2007 Mar 14;2(3):e282. doi: 10.1371/journal.pone.0000282 (PMC1804098; doi:10.1371/journal.pone.0000282)
Supplement: Mathematical Details S1 — Here we give more details and references about the individual-level models presented in the main text. We also briefly discuss how the concept of right censoring manifests in our simulations. (0.05 MB PDF) [file pone.0000282.s001.pdf]

Theory versus Data: How to calculate R_0?

MATHEMATICAL DETAILS S1

Romulus Breban[*], Raffaele Vardavas[*] & Sally Blower[†]

The Semel Institute of Neuroscience and Human Behavior, David Geffen School of Medicine at University of California Los Angeles, 1100 Glendon Avenue PH2, Los Angeles, CA 90024

Branching processes assume that the individual-level processes are independent and identically distributed. Moreover, in a branching process, individuals statistically follow the same course of events. The most common branching process in Epidemiology is the Crump-Mode-Jagers process (CMJ) [1-7], which is a continuous time branching process where each individual follows a stochastic scenario.[‡] Certain CMJ processes are compatible with ODE models of disease invasion that describe the initial phase of exponential growth of an epidemic.[§] (See Fig. 3A for an example of numerical agreement between a CMJ process and an ODE model of disease invasion.) R_0 is conceptually clear when CMJ branching processes are applied to population-level data [5,

[*] Romulus Breban and Raffaele Vardavas contributed equally to the research described in this paper.
[†] To whom correspondence should be addressed. E-mail: sblower@mednet.ucla.edu
[‡] For example each individual remains infectious for a time interval exponentially distributed with average μ^{-1} while infecting other individuals such that the time intervals between infections are also exponentially distributed with average β^{-1}.
[§] The work of Crump, Mode, and Jagers has been further developed by Ball and Donnelly [8] who constructed a generalized CMJ process with a finite number of susceptibles that converges to the ODE *SIR* model. In this case, the value of R_0 can be calculated analytically and shown to be equal to the threshold value obtained by analyzing the ODE *SIR* model.

9]. Given the individual-level course of events of a CMJ process, the expected R_0 can be easily calculated. In analyzing contact tracing data (assumed to be described by a CMJ process) or computer simulations of CMJ processes, one averages the number of secondary infections produced by individuals who are no longer infectious. However, in calculating these statistics, one realizes that individuals who were recently infected but are no longer infectious are not representative, since many of their peers infected at the same time are still infectious. This statistical bias is known as *right censoring* [10], and it is justified by the fact that every individual undergoes the same course of events and that the expected R_0 calculated for a single individual must match the value obtained by averaging over the population.

For concreteness, let us consider the case of the *SI* ODE model at disease invasion; i.e., the *SI* ODE applied to the exponential growth phase of an epidemic. Mathematically, the R_0 of the corresponding CMJ process is β/μ [5]. This is confirmed by the numerics in Fig. 3B (open circles) that simulate contact tracing; we obtain a plateau value at β/μ in the number of secondary infections. Right censoring manifests as a smooth decline in the number of secondary infections from the plateau value.

Our ILM consists of a growing network of infectious individuals where two individuals are linked if one infected the other. New links are added to the network according to a population-level rate of β per infectious capita. The new links are randomly assigned to the individuals in the network. Infectious individuals are removed from the network at a population-level rate μ per infectious capita according to a removal rule. Assuming that the number of secondary cases an infectious individual has caused is a proxy for the progression of the disease, we choose a removal rule that favors the removal of individuals with large number of secondary infections. The probability of an infectious individual being removed is taken to be proportional to his/her number of secondary infections plus one. We proceed similarly to the case of the CMJ model, counting the number of secondary infections of each removed individual and stratifying it versus their time of infection. Figure 3B (dots) shows the corresponding numerics. We

find a plateau below β/μ that ends with a smooth decline due to right censoring.[**] Therefore, R_0 in this case is less than β/μ, although the population dynamics has an epidemic threshold parameter of β/μ.

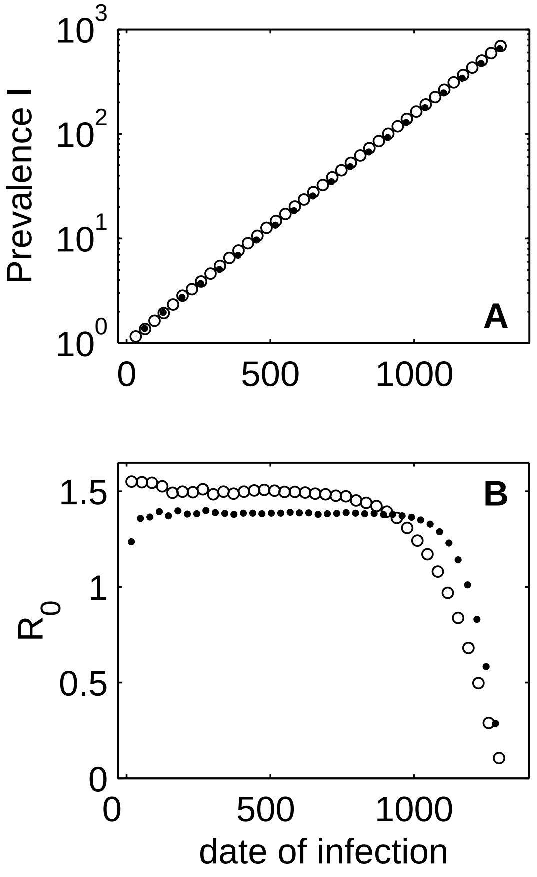

Figure 3. Comparison plots between our example ILM and branching process. **A** The average prevalence versus time. The open circles represent results for the branching process while the dots represent results for our ILM. We used $\beta = 0.015$, $\mu = 0.01$, and we averaged over 1,000 stochastic realizations. On a logarithmic scale, the results fit very well with a straight line with slope $(\beta - \mu)$ and intercept 0 that corresponds to the ODE solution $I(t)=I(0)exp[(\beta-\mu)t]$, where $I(0)=1$. **B** The average number of secondary infections stratified versus the date of infection. The open circles represent results for the branching process while the dots represent results for our ILM. We used $\beta = 0.015$, $\mu = 0.01$, and we averaged over 15,000 stochastic realizations. The R_0 of the branching process is 1.5, while the R_0 of our ILM is approximately 1.4.

[**] For CMJ processes right censoring is well-defined due to the fact that every individual undergoes the same stochastic scenario which generates an expected R_0. However, for a more general ILM that does not have this feature, right censoring cannot be consistently defined [11].

References

1. Crump, K. and C. Mode, *A general age-dependent branching process I.* Journal of Mathematical Analysis and Applications, 1968. **24**: p. 494-508.
2. Crump, K. and C. Mode, *A general age-dependent branching process II.* Journal of Mathematical Analysis and Applications, 1969. **25**: p. 8-17.
3. Mode, C., *Multitype branching processes-theory and applications*. 1971, New York: American Elsevier.
4. Mode, C., *Stochastic processes in demography and their computer implementation*. 1985, Berlin: Springer-Verlag.
5. Mode, C. and C. Sleeman, *Stochastic processes in epidemiology*. 2003, Singapore: World Scientific.
6. Jagers, P., *A general stochastic model for population development.* Skandinavisk Aktuarietidskift, 1969: p. 84-103.
7. Jagers, P., *Branching processes with biological applications*. 1975, London and New York: John Wiley and Sons.
8. Ball, F. and P. Donnelly, *Strong approximations for epidemic models.* Stochastic Processes and Applications, 1995. **55**: p. 1-21.
9. MacDonald, G., *The analysis of equilibrium in malaria.* Tropical Diseases Bulletin, 1952. **49**: p. 813-829.
10. Bootsma, M.C.J. and O. Diekmann, *Comment on 'Linking population-level models with growing networks: A class of epidemic models'.* Physical Review E, 2006. **74**(1): p. 018101.
11. Breban, R., R. Vardavas, and S.M. Blower, *Reply to "Comment on 'Linking population-level models with growing networks: A class of epidemic models' ".* Physical Review E, 2006. **74**(1): p. 018102.
